# Supplementary material for: Evidence supported by Mendelian randomization: impact on inflammatory factors in knee osteoarthritis
Source: Front Med (Lausanne). 2024 May 28;11:1382836. doi: 10.3389/fmed.2024.1382836 (PMC11165061; doi:10.3389/fmed.2024.1382836)
Supplement: Supplementary file 1 [file Data_Sheet_1.ZIP › data/GCST90274808_5e6/808.forest.pdf]

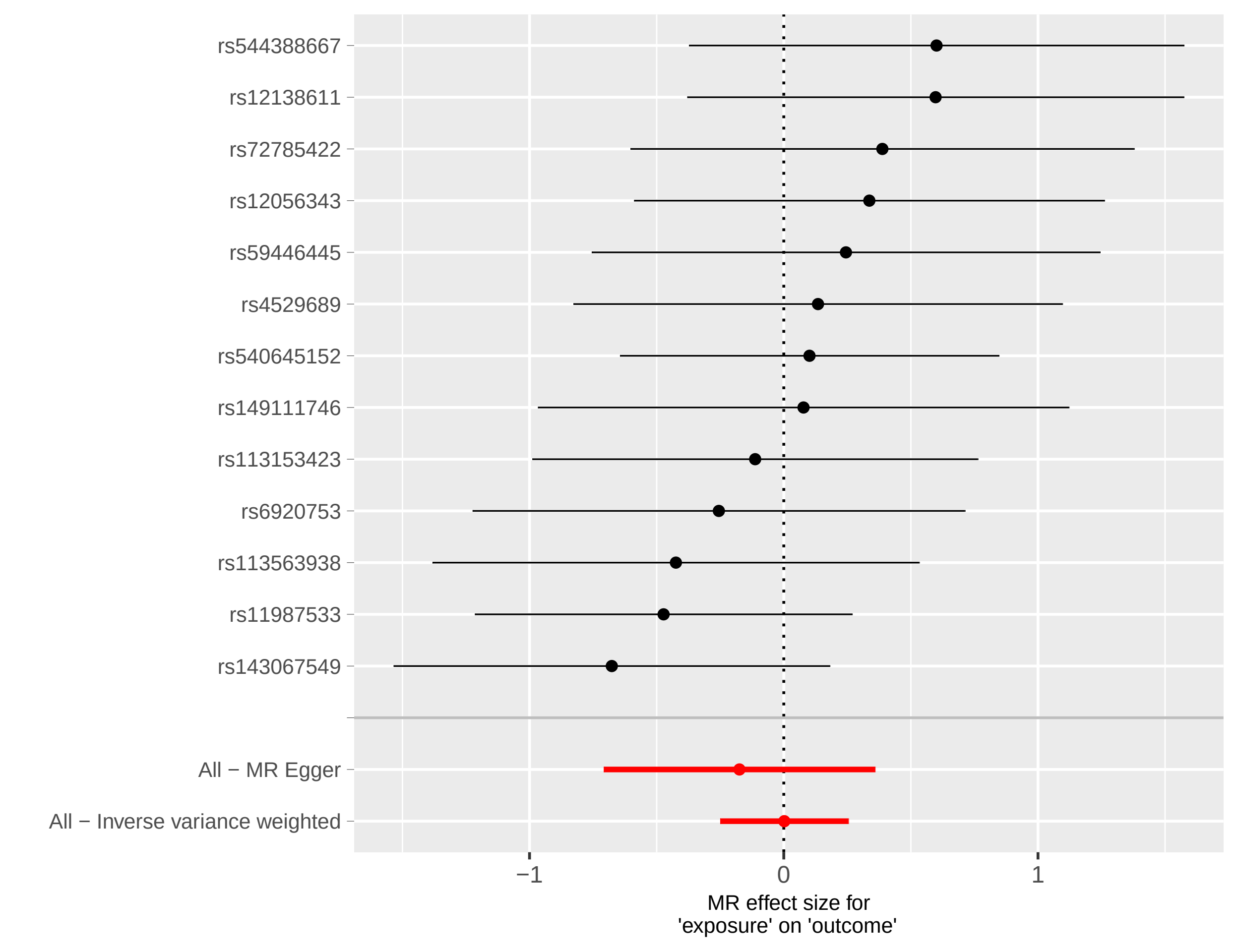

rs544388667
rs12138611
rs72785422
rs12056343
rs59446445
rs4529689
rs540645152
rs149111746
rs113153423
rs6920753
rs113563938
rs11987533
rs143067549
All − MR Egger
All − Inverse variance weighted
−1
0
1
MR effect size for
'exposure' on 'outcome'
